# Citizen science project characteristics: Connection to participants’ gains in knowledge and skills

**DOI:** 10.1371/journal.pone.0253692

**Published:** 2021-07-15

**Authors:** Maria Peter, Tim Diekötter, Kerstin Kremer, Tim Höffler

**Affiliations:** 1 Department of Landscape Ecology, Institute for Natural Resource Conservation, Kiel University, Kiel, Germany; 2 IPN—Leibniz Institute for Science and Mathematics Education, Kiel, Germany; 3 Biology Education, IDN—Institute for Science Education, Leibniz University Hannover, Hannover, Germany; Instituto Federal de Educacao Ciencia e Tecnologia Goiano - Campus Urutai, BRAZIL

## Abstract

**Background:**

Biodiversity is being lost rapidly and its conservation is thus one of the most urgent tasks today. For biodiversity conservation to be successful, the public needs to gain an awareness and understanding of biodiversity and its importance. Moreover, species experts are needed who have the skills necessary for identifying and recording biodiversity. Previous research showed that citizen science projects can contribute to educating the public about biodiversity. However, it is still unclear how project characteristics connect to participants’ knowledge and skills and how citizen science projects should be designed if they are to foster participants’ learning.

**Aim:**

We aimed to investigate specific characteristics of biodiversity citizen science projects that could potentially influence participants’ learning. We explored the following project characteristics from both the project coordinators’ and the participants’ perspectives: information and training provided to participants, social interaction among participants, contact between participants and staff, and feedback and recognition provided to participants.

**Methods and results:**

In order to examine the extent to which these project characteristics are connected to participants’ gains in knowledge and skills, we conducted a comprehensive study across 48 biodiversity citizen science projects in Europe and Australia. We found that participants’ perceived gains in knowledge and skills were significantly related to the five project characteristics as reported by the participants: information received by the participants, training received by the participants, social interaction among participants, contact between participants and staff, and feedback and recognition received by the participants.

**Conclusion:**

We conclude that by deliberately designing citizen science projects to include features such as interaction and feedback, these projects could achieve higher learning outcomes for the participants. Thereby, suitable modes of communication between projects and their participants are crucial. We provide specific suggestions for the design of biodiversity citizen science projects and for future research on project characteristics and participant outcomes.

## Introduction

### Citizen science and biodiversity conservation

Biodiversity, the diversity of life on Earth, is essential for functioning ecosystems and, therefore, ultimately for human well-being [1, 2]. Biodiversity, however, is disappearing rapidly [3, 4]. In order for biodiversity conservation to become a priority, it is essential to raise public awareness and understanding of the concept of biodiversity [5], the importance of biodiversity [6, 7], and the threats posed to biodiversity [5].

In addition to an awareness and understanding of biodiversity, an ability to recognize and identify biological diversity, for example, on the level of species, is crucial [5]. "Species experts" are needed who are able to identify, monitor, and record biodiversity [8, 9]. The number of such experts, be they amateurs or professionals, has steadily declined in the past decades [8, 10, 11]. In addition, students of different ages [9, 12] as well as biology teachers [8, 13] have been found to have poor knowledge of species. This is not surprising as the number of university courses that teach species identification has been declining [14]. As a result, biology graduates often have little knowledge about species and insufficient skills of species identification [8]. Consequently, less knowledge and fewer skills can be passed on to students. In order to reverse this "erosion of species experts" [8] and the decline in skills and knowledge, other ways of fostering relevant knowledge and skills outside of, or in addition to, formal education curricula are needed. In this regard, citizen science (CS) projects focusing on biodiversity could be a way forward [15–17].

Citizen science is defined as public participation in scientific research [18] or community-based monitoring. CS projects are authentic research projects that involve members of the general public (*citizens*) in the research process [19, 20]. Such projects have become particularly popular in a biodiversity-related context. In this context, Theobald et al. [21] defined biodiversity as "the presence and/or abundance of identified taxonomic (e.g., species, genus, family), genetic, or functional groups" (p. 237). Biodiversity citizen science (BDCS) projects involve the general public in identifying and monitoring biological diversity and collecting biodiversity-related data [21]. BDCS projects have thereby made it possible for professional scientists to collect large amounts of data that they would otherwise not have been able to obtain [21–23]. By involving citizens in the research process, BDCS has thus contributed considerably to biodiversity research (for reviews see, e.g., Donnelly et al. [24], Theobald et al. [21], Chandler et al. [25], Irga et al. [26], Heilmann-Clausen et al. [27]).

### Project participants’ gains in knowledge and skills

In addition to scientific outcomes, many BDCS projects seek to achieve participant outcomes. Participant outcomes are CS project outcomes for the individual participating citizens, as described by Jordan et al. [28] and Shirk et al. [29] and defined in detail by Phillips et al. [30]. Participant outcomes can, for example, be gains in knowledge and skills, increased interest, motivation, and self-efficacy, as well as changes in behavior [17, 30] and other personal outcomes [16, 17, 31].

Gains in knowledge and skills are among the participant outcomes most often sought by CS projects [30, 32]. Knowledge and skills were defined by Phillips et al. [30] as follows (p. 7 and 9):

- Knowledge: "Knowledge of science content (i.e., understanding of subject matter) and the nature of science; understanding of the scientific process and how science is conducted"
- Skills: "Procedural skills such as asking questions, designing studies, collecting, analyzing, and interpreting data, experimenting, argumentation, synthesis, technology use, communication, and critical thinking"

Several case studies found an increase in participants’ knowledge [33–41] and skills [33, 37, 39, 40, 42]. A recent comprehensive study across a variety of BDCS projects found gains in participants’ environmental and scientific knowledge as well as scientific skills [17].

### Citizen science project characteristics

While research indicates that participation in BDCS projects can contribute to participants’ learning about biodiversity, it would be useful to know how BDCS projects have to be designed in order to achieve such gains in participants’ knowledge and skills. Various authors have emphasized the necessity for research into such characteristics or design features of CS projects [17, 30, 39, 43–45]. Existing literature on CS project characteristics largely focuses on how these characteristics are associated with general project success [32, 46–51], which is often synonymous with participant motivation and retention. In addition, several authors have discussed CS project characteristics that could potentially affect participants’ learning in general. Project characteristics that are most often mentioned in this context are: information provided to the project participants [40, 44, 52–55], training provided to the participants [39, 44, 55, 56], social interaction among participants [44, 53, 57–61], contact between project staff or scientists and project participants [55, 62–66], and feedback and recognition provided to the participants [36, 44, 53, 55, 66–68].

While the literature suggests that such project characteristics can positively affect participants’ learning on the whole, actual studies on specific learning outcomes such as gains in knowledge or skills, and how these are influenced by the CS project’s design, are scarce. Gains in participants’ scientific knowledge [69, 70] and environmental knowledge [71] have been investigated in connection with participants’ training only. Gains in scientific skills have been investigated in connection with individual feedback provided to participants [72] and social interaction among participants [15]. While these few case studies of individual CS projects are valuable, their findings are not comprehensive enough to facilitate conclusions about the design of BDCS projects. Indeed, various authors recommended more comprehensive and comparative studies that are conducted across several CS projects (e. g., [30, 31, 73, 74].

### Aims and research question

In this study, we addressed this scarcity of research that links specific project characteristics to participant outcomes of BDCS projects. We conducted an exploratory study that focused on the following research question:

*To what extent are participants’ gains in knowledge and skills connected to the following project characteristics*?

- *Information provided to participants*
- *Training provided to participants*
- *Opportunities for social interaction among participants*
- *Contact between project participants and project staff*
- *Feedback and recognition provided to participants*

To address this research question, we adopted a large-scale approach and conducted a study across various BDCS projects taking place in several countries. In order to get a comprehensive overview, we investigated the perspective of both the project staff/coordinators and the project participants/volunteers in our study. To the best of our knowledge, we are the first to conduct this kind of comprehensive study across projects and countries, including project coordinators as well as participants.

## Methods

### Overview of the study

Our study comprised two surveys:

1. An online survey of BDCS projects. This survey addressed the project coordinators. The aim of the survey was to gather comprehensive and detailed information about BDCS projects and how they are designed, managed, and conducted. We will refer to this survey as the *coordinator survey* throughout the article.
2. An online survey of BDCS project participants. This survey was aimed at adults participating in BDCS projects as volunteers. The aim of this survey was to obtain information about the participants’ view of the project and their perceived gains in, for example, knowledge and skills. We will refer to this survey as the *participant survey*.

These online surveys were administered to coordinators and participants of BDCS projects in Europe and Australia; the responses were analyzed quantitatively. We focused on these two regions, first, for language reasons, and second, because of the high number of BDCS projects that existed in these regions. We did not include North America because previous studies were mainly conducted in that region [31].

### Citizen science projects taking part in the study

We systematically searched for suitable BDCS projects in various project databases provided by, for example, the Australian Citizen Science Association [75] (https://citizenscience.org.au/ala-project-finder), Österreich forscht [76] (Austria, https://www.citizen-science.at/aktuelleprojekte), Bürger schaffen Wissen [77] (Germany, https://www.buergerschaffenwissen/projekte), and SciStarter [78] (global, http://scistarter.com). We chose BDCS projects according to the definition by Peter et al. [31]:

1. CS projects that involve volunteers in monitoring and identifying biological diversity and collecting biodiversity data [21]. We excluded CS projects that were only indirectly related to biodiversity, for example, projects focusing on water quality or birds’ nesting success.
2. Nature- or field-based CS projects that take place outdoors. They include observation of or interaction with nature. In addition, these projects often involve online activities such as species identification or data submission, but they are not limited to such online activities. We excluded projects that did not ask their participants to observe or interact with nature, for example, exclusively online projects that invited volunteers to identify species in online photo databases (see Aristeidou and Herodotou [45] for more information on online CS projects).

**Fig 1:**
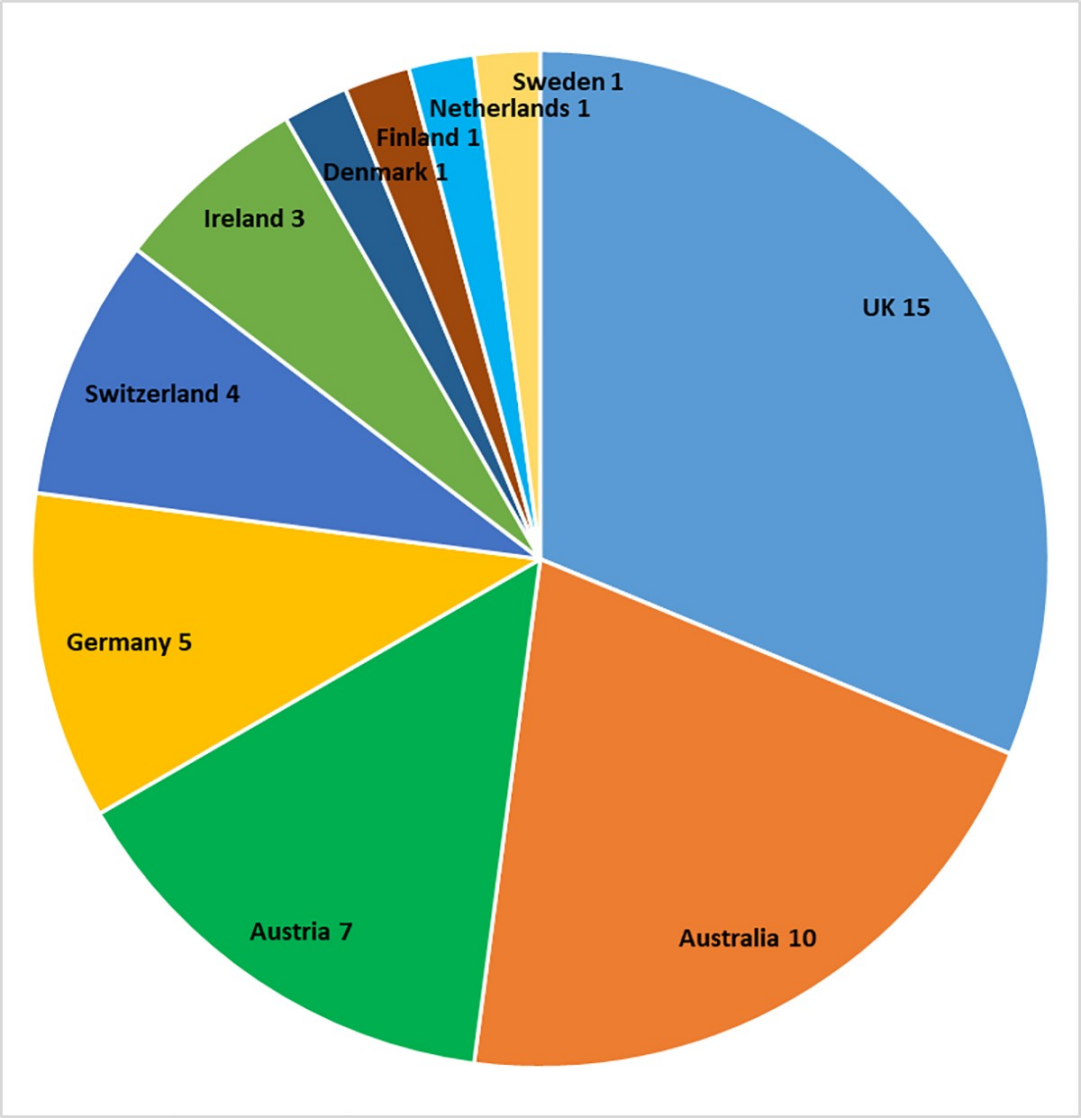
Number of BDCS projects in each country (total number: 48).

Our study included 48 BDCS projects from 10 different countries, the country with the highest number of participating projects being the UK (15 projects), followed by Australia (10), Austria (7), and Germany (5) (Fig 1). The 10 projects with the highest number of volunteers taking part in the participant survey were Garden Bird Watch (UK), Wild Pollinator Count (Australia), Breeding Bird Survey (UK), Kerbtier.de (Germany), NaturTjek (Denmark), Irish Garden Bird Survey (Ireland), NABU|naturgucker (Germany), UK Wetland Bird Survey (UK), Meetnet Vlinder (Netherlands), and Schmetterlinge Österreichs (Austria). For a complete list of the 48 projects taking part in our study, please see S1 Table in S1 File.

**Fig 2:**
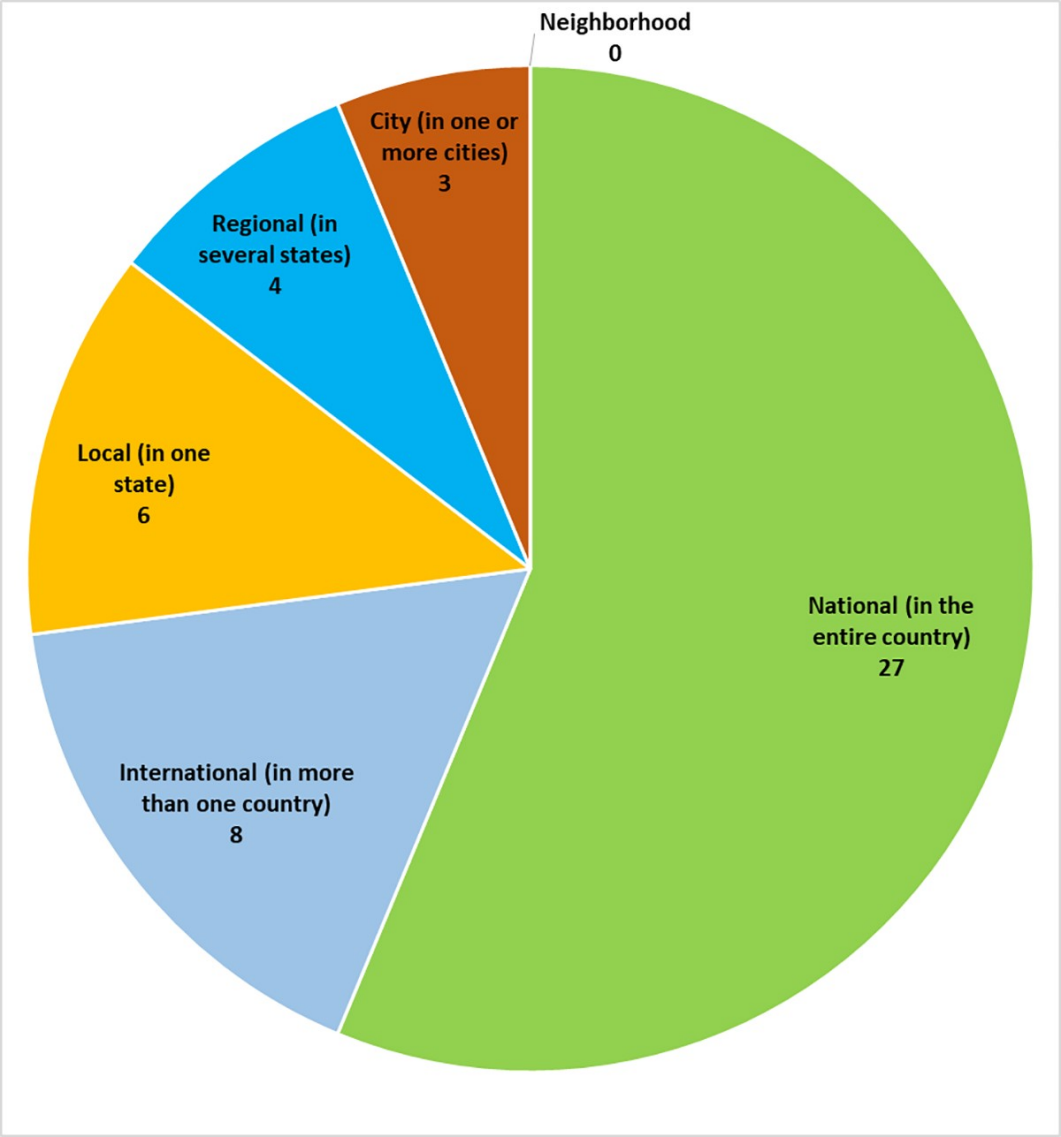
Geographic scope of participating projects.

**Fig 3:**
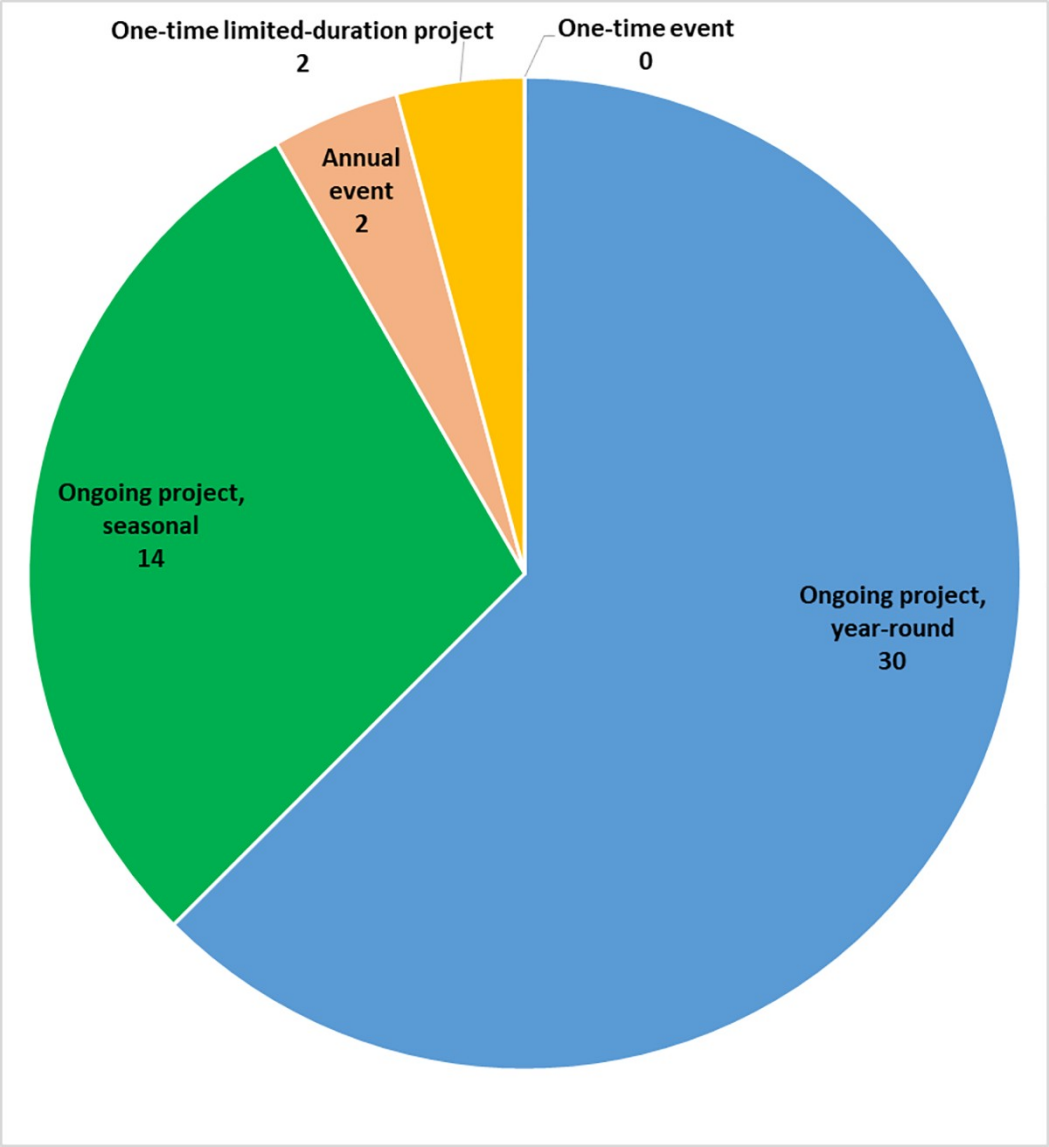
Time frame of participating projects.

**Fig 4:**
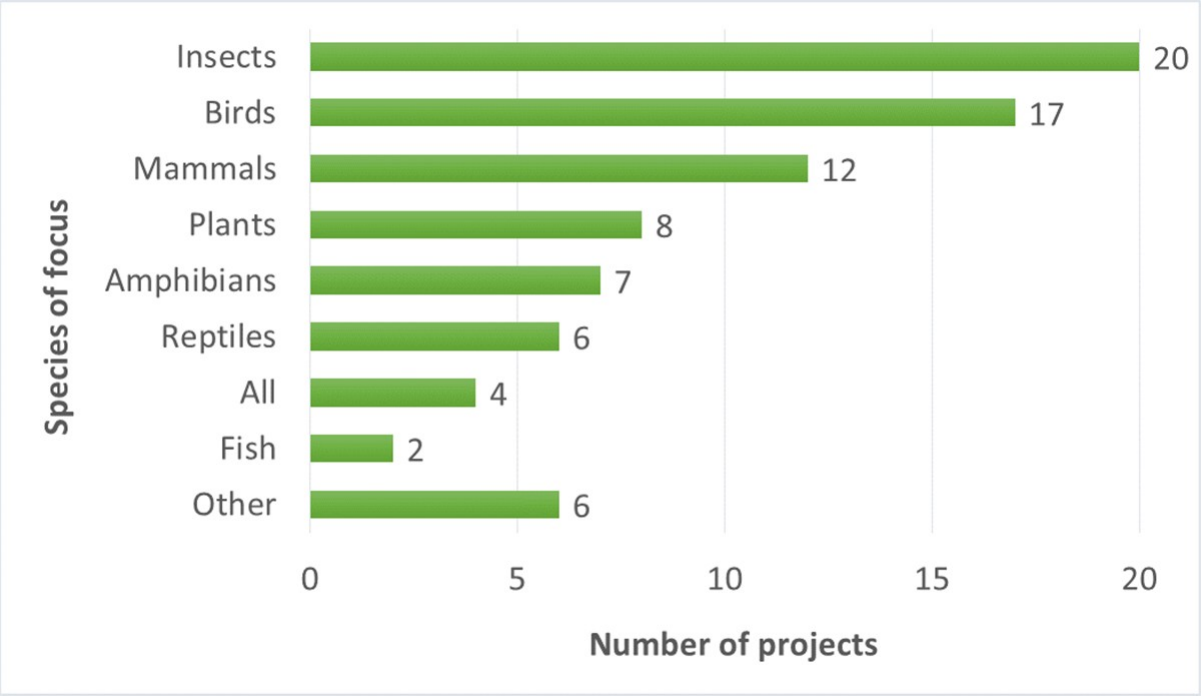
Species or organisms that are the focus of the participating projects (multiple answers possible).

**Fig 5:**
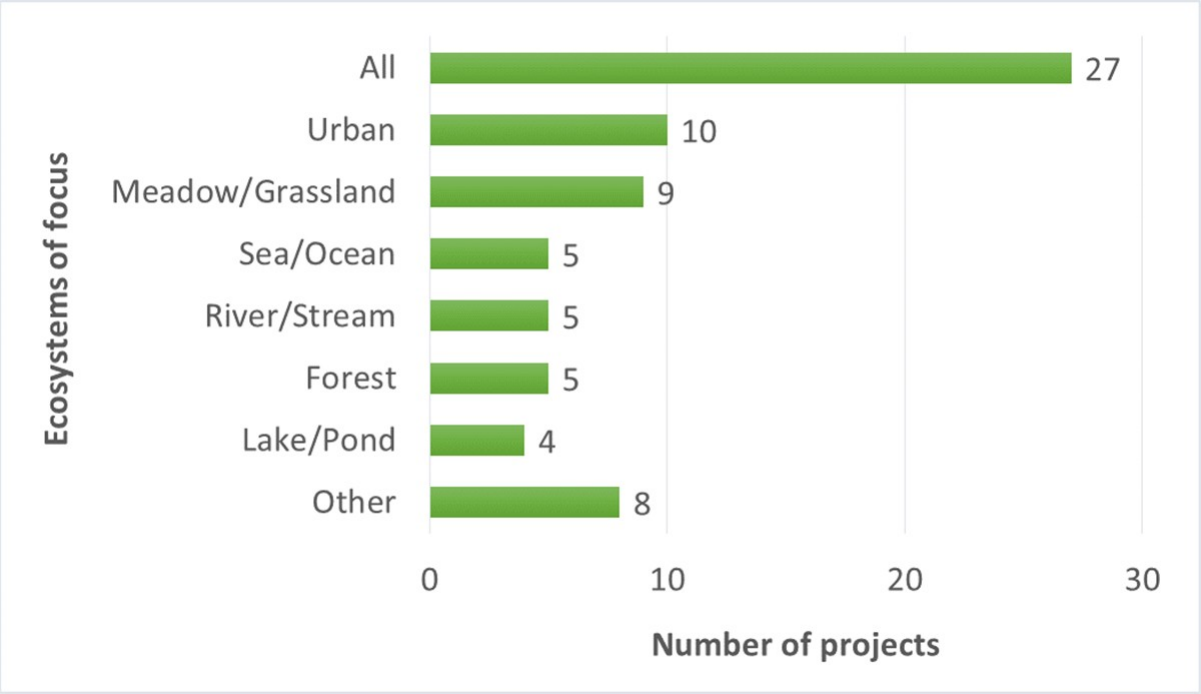
Ecosystems that are the focus of the participating projects (multiple answers possible).

The geographic scope of most BDCS projects in our study was nationwide (Fig 2). The majority of projects were ongoing projects taking place either year-round or seasonally (Fig 3). The participating projects largely focused on specific organisms such as insects, birds, and mammals (Fig 4), but a few projects focused on a particular ecosystem (Fig 5).

### Citizen science project participants

The demographic characteristics of the project participants were diverse (Table 1). While all ages were represented, slightly more than half of the participants were at least 60 years old (53%). Men (47%) and women (54%) were nearly equally represented. Most participants had at least a bachelor’s degree (68%). The majority of participants were either working (48%) or retired (45%).

**Table 1:** Demographic characteristics of project participants who took part in the study.

Demographic characteristics (n = 838)		Respondents (%)
**Age**	18–29 years	3.3
** **	30–39 years	5.8
** **	40–49 years	13.7
** **	50–59 years	23.9
** **	60–69 years	36.3
** **	70 years and over	16.9
**Gender**	Female	52.6
** **	Male	47.3
** **	Other	0.1
**Education**	Some high school	3.6
** **	Completed high school	8.7
** **	Completed technical or vocational training	19.8
** **	Bachelor’s degree	27.9
** **	Master’s or other postgraduate degree	29.6
** **	Doctorate (PhD, EdD, MD, etc.)	10.5
**Employment**	Employed full- or part-time	36.4
** **	Self-employed	11.2
** **	Full-time housewife/-husband	1.8
** **	Out of work	3.6
** **	Student	1.9
** **	Retired	45.0

Most participants had been involved in their CS project for one year or more (88%), some of whom had been active for over 10 years (21%), mostly by identifying and recording species and submitting those data to the project. The majority of participants had been actively involved in the project in the last month before their participation in the online survey (64%). Most participants spent more than 10 hours per year on project activities (59%).

### Development of the surveys

We developed both surveys by analyzing and comparing questionnaires that had already been used in previous studies. Survey questions in the coordinator survey were informed by questions found in various existing questionnaires [30, 53, 79]. Similarly, the survey questions in the participant survey were inspired by questions found in previously used questionnaires, for example, by Toomey and Domroese [65], Chase and Levine [80], and Lewandowski and Oberhauser [54]. For the participant survey questions concerning skills, we adapted the Skills for Science Inquiry Scale [81] provided by the Cornell Lab of Ornithology. We slightly shortened the scale in order to reduce the length of the questionnaire. In addition, we changed the wording of some items to make them more suitable for the context of our study. We selected suitable items for inclusion in the two surveys based on theoretical relevance. We then adapted the content and wording of these items to the subject and aims of our study.

We developed both an English and a German version of the questionnaires. Both questionnaires were originally created in English, in order to facilitate the international approach of our study across countries. The questionnaires were then translated into German, reflecting the local setting of our research team, which is based in Germany. The translation was done by two independent translators who were not part of the research team. We then chose the most suitable translations. In order to assure that the questions were clear and easy to understand, and to determine the average time required to complete the surveys, both questionnaires were pre-tested: the coordinator questionnaire was pre-tested by five CS project coordinators and seven members of the general public with a background in natural or social sciences; the participant questionnaire was pre-tested by 45 members of the general public of different ages and with different educational as well as professional backgrounds (36 in German, nine in English). Respondents’ feedback and suggestions for improvement were then integrated into the final versions of both questionnaires. In order to ensure the validity of the scales, we based our survey questions on pre-existing scales and on items found in questionnaires previously used by other authors.

### Coordinator and participant questionnaires

The coordinator questionnaire addressed project characteristics from the perspective of the project staff and organizers. The coordinator questionnaire contained the following sections: general information about the project, project goals and outcomes, project activities, requirements of and training for participants, information provided to participants, opportunities for social interaction among participants, contact between project participants and project staff, and feedback and recognition provided to participants. An example of a questionnaire item regarding information provided to participants is shown in Table 2. All items concerning training, information, social interaction, contact, and feedback and recognition are available in S2–S6 Tables in S1 File. The complete coordinator questionnaire is available upon request.

**Table 2:** Example of an item in the coordinator questionnaire concerning information provided to project participants.

Do you provide your participants with information about the following?								
	No	Yes, on the project website	Yes, through social media	Yes, mess-ages through the project app	Yes, through emails	Yes, through mailings (paper)	Yes, in training sessions	Other
Overall objectives / goals / intended outcomes of your project								
Scientific background and processes of your project								
Overall results / outcomes of your project								
Threats to the species that your project focuses on								
Opportunities for engaging in conservation activities outside your project								

The participant questionnaire addressed project characteristics from the participants’ perspective, as well as perceived participant outcomes. The participant questionnaire comprised the following sections: questions about the project, about the respondents (demographics), about the amount and nature of participation, and about perceived outcomes. These self-reported outcomes concerned perceived changes in, for example,

- participants’ knowledge: awareness of species, understanding of biodiversity, learning about species, nature, and science; and
- participants’ skills: data collection skills, further skills such as data analysis, and so forth

The questionnaire items concerning information, training, social interaction, contact, and feedback and recognition are available in S7–S11 Tables in S1 File. The items for self-reported changes in knowledge and skills are available in S12 and S13 Tables in S1 File. The complete participant questionnaire is available upon request.

Both questionnaires contained mostly closed-ended questions (e.g., 5-/6-point Likert-type, multiple-choice) as well as a smaller number of open-ended questions providing an opportunity for additional comments. The closed-ended questions were mandatory; the open-ended questions were optional. While the coordinator questionnaire included project name, organization, and contact details, the participant questionnaire was anonymous. Informed consent was obtained from the participants at the start of the online survey. The survey was approved by the Ethics Committee of the Leibniz Institute for Science and Mathematics Education.

### Conducting the surveys

The coordinator survey was administered to project coordinators directly. In the large CS projects, project coordinators often were staff members of the organization or institution responsible for the project. In the smaller projects without paid staff, project coordinators were volunteers. In some cases, the survey was completed by other project staff (e.g., former project coordinator on parental leave). Unlike the coordinator survey, the participant survey could not be administered directly to project participants due to privacy concerns and data protection issues. Instead, project coordinators invited project participants to take part in the survey, either through an invitation email or by including the link to the survey in a project newsletter or by posting a message on the project website.

Both surveys were conducted online, using LimeSurvey software, version 3.17 and 3.23. The coordinator questionnaire was open from October 8, 2019 to October 1, 2020 and was (at least partly) filled in by 56 project coordinators or their staff. Out of the 56 coordinator responses, only 48 projects had corresponding participant responses. The participant questionnaire was open from July 4, 2019 to November 30, 2019. During that time, LimeSurvey registered 1,179 survey respondents who provided at least their country and the name of the CS project they were participating in. The questionnaire was filled in by participants of the projects that were taking part in our study, as well as by participants of other projects. The dataset derived from the participant survey was analyzed in a previous study focusing on a variety of participant outcomes (see [17]).

For the current study, we combined both the coordinator and the participant datasets. In this combined dataset, we only kept participants’ responses for which we had a corresponding coordinator response, and vice versa. Consequently, our combined dataset contained 48 coordinator responses (of which 47 were completed, one was partly filled in) and 1,067 participant responses (837 completed).

### Statistical analysis of survey data

We analyzed the quantitative data of the combined dataset using IBM SPSS Statistics software, version 26. For responses to 5-point Likert-type questions we assigned the values 1 to 5 (e.g., responses coded as 1 for *strongly disagree* to 5 for *strongly agree*). In a previous study, participant outcomes such as gains in knowledge and skills were investigated [17]. In that study, single items concerning participants’ perceived gains in knowledge and skills were combined into scales. In the current study, we used the same scales (see Table 3).

**Table 3:** Overview of scales for knowledge and skills (Peter et al. 2021).

Scales	Number of items	Reliability (Cronbach’s Alpha)
**Knowledge**		
Change in awareness of species	3	0.857
Change in understanding of biodiversity	3	0.951
Learning about species, environment, and science	3	0.769
**Skills**		
Gain in skills of data collection	4	0.845
Gain in skills of data analysis, etc.	6	0.865

First, we investigated whether and to what extent participants’ perceived changes in knowledge and skills were related to different project characteristics. We investigated the following project characteristics from the perspective of both project coordinators and project participants:

- Information provided by the project / received by participants
- Training provided by the project / received by participants
- Opportunities for social interaction among participants as provided by the project / used by participants
- Opportunities for contact between project participants and project staff as provided by the project / used by the participants
- Feedback and recognition provided by the project / received by participants

We performed one-way independent analyses of variance (ANOVAs, including Levene’s test of variance and Welch’s test whenever equal variance could not be assumed). In addition, we summarized some variables of the project characteristics and examined whether these summary variables correlated with perceived changes in knowledge and skills. For this purpose, we calculated the Pearson correlation index.

In the following section, we present the results of the quantitative data analyses. We provide the number of respondents who answered a question (sample size, *n*), averages (mean, *M*), the spread of data around the mean (standard deviation, *SD*), the significance of results (*p*, results are statistically significant if *p* < 0.05), and the size of the observed effect (partial eta squared, η^2^, and Pearson’s correlation coefficient *r*) (see Field [82]. For effect sizes, we followed the widely used suggestions by [83], who defined effect sizes as follows:

- Partial eta squared: small effect: partial η^2^ ≥ 0.01, medium effect: partial η^2^ ≥ 0.06, large effect: partial η^2^ ≥ 0.14.
- Pearson correlation index: small effect: *r* ≥ 0.1, medium effect: *r* ≥ 0.3, large effect: *r* ≥ 0.5.

When we mention a *majority* of respondents, we are referring to a proportion of survey respondents greater than 50%.

## Results

### Participants’ perceived gains in knowledge and skills

In the participant survey, we asked project participants to report perceived changes in knowledge and skills resulting from project participation. Survey respondents answered by choosing their level of agreement with a specific statement regarding gains in knowledge and skills.

#### Gains in knowledge

The majority of the participant survey respondents agreed that, as a result of participating in their project, they had become more aware of species’ presence, diversity, and threats to these species (Table 4). Similarly, many respondents agreed that participating in the project had increased their understanding of biodiversity and its importance, and threats to biodiversity. Finally, participants agreed that, through participating in the project, they had learned a lot about the species they found or observed, about the environment in general, and, to a lesser degree, about how science works.

**Table 4:** Scales for self-reported changes in participants’ knowledge and skills.

Scale	*n*	Mean*	*SD*
**Knowledge**	** **	** **	** **
Change in awareness of species	871	4.13	0.73
Change in understanding of biodiversity	871	3.67	0.95
Learning about species, environment, and science	871	3.50	0.77
**Skills**			
Gain in skills of data collection	859	3.87	0.70
Gain in skills of data analysis, etc.	859	2.89	0.73

(*1 = Strongly disagree, 2 = Disagree, 3 = Neutral, 4 = Agree, 5 = Strongly agree).

#### Gains in skills

Most respondents of the participant survey agreed that, as a result of participating in the BDCS project, they had gained or increased their data collection skills (Table 4) such as observing and recording species, identifying different species, collecting data in a standardized manner, and submitting their observations to the project database. Fewer respondents agreed that their skills beyond data collection had also increased through project participation. Such skills were: using the project database to answer a question, communicating project findings to others, interpreting the meaning of project data presented in maps and charts, training others to participate in the project, conducting statistical analyses using project data, and designing their own study related to project data.

### Project characteristics

We analyzed the extent to which gains in knowledge and skills reported by project participants were related to the following project characteristics:

- Information provided to and received by participants
- Training provided to and received by participants
- Opportunities for social interaction among participants
- Contact between project participants and project staff
- Feedback and recognition provided to and received by participants

We analyzed both the perspective of the project coordinators (e.g., training *provided* by the project, as reported by the respondents to the coordinator questionnaire) and the perspective of the participants (e.g., training *received* by the participants, as reported by the respondents to the participant questionnaire). We found statistically significant relationships between project characteristics and participant outcomes for all five project characteristics. In most cases, these results were statistically significant for project characteristics as described by the participants, but not for project characteristics as described by the coordinators. Here, we report only statistically significant results (*p* < 0.05) with at least small effect sizes (partial η^2^ ≥ 0.01, or *r* ≥ 0.1). Significant results with less than small effect sizes are not reported.

#### a. Information provided to and received by participants

**Fig 6:**
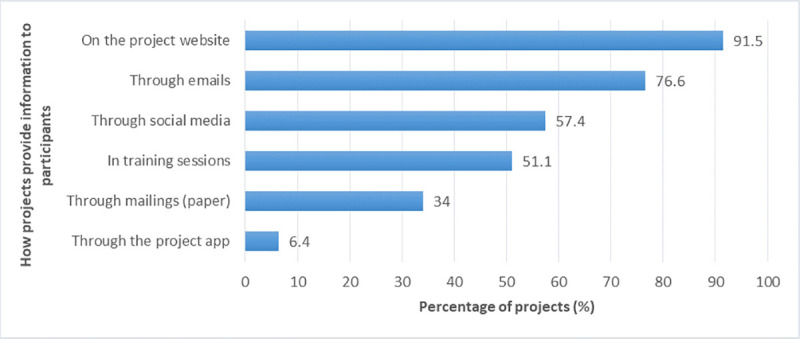
Means of providing information to the project participants.

In the coordinator survey, we asked the BDCS project coordinators what information they provided their participants with and how they provided the information (multiple-choice questions). Most projects provided information on project goals/intended outcomes (96%) and project results (94%), followed by information about threats to the species that the project focused on (89%), the scientific background and processes of the project (89%), and opportunities for engaging in conservation activities outside the project (68%). The means of providing information used most frequently were the project website (92% of projects), followed by emails (77%) (Fig 6).

**Fig 7:**
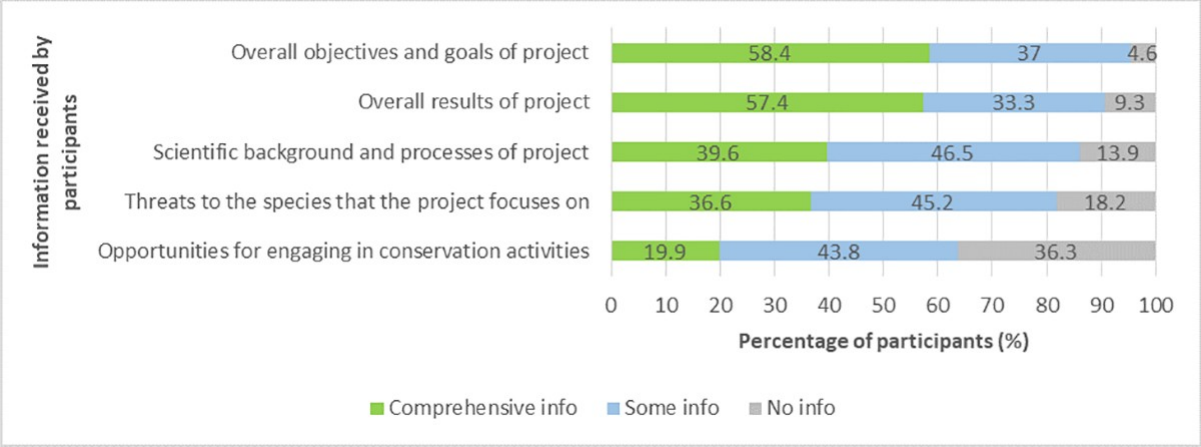
Kinds of information received by the participants.

Similarly, in the participant survey, we asked the project participants about the information they received from their project (possible answers: no information, some information, comprehensive information). The majority of respondents stated that they received comprehensive information about the project’s goals (58%) and overall results (57%) (Fig 7). Project newsletters or other information received from the project were read by participants at least once a month (36%), less than once a month (59%), or never (5%).

The analysis of the participant data revealed that participants’ perceived gains in both knowledge and skills were significantly related to the amount of information that participants received regarding the project’s scientific background and overall results, the threats to the species the project focused on (Table 5), the opportunities for engaging in conservation activities outside the project, and partly to the information they received about the project’s overall goals (learning about species, etc., skills of data analysis, etc.); effect sizes were small in all cases. Participants who received more information reported higher gains in knowledge and skills. In addition, participants who read such information more frequently reported higher gains in knowledge and skills; effect sizes were small for skills and small to medium for knowledge.

**Table 5:** Results of the one-way independent ANOVA test for the amount of information about threats to species that was received by the project participants.

Scale	*df* (Degrees of freedom)	*F*	*p* (Significance)*	Partial η^2^ (Effect size)**
**Knowledge (*n* = 871)**				
Change in awareness of species	2, 868	19.569	**<0.001**	0.043
Change in understanding of biodiversity	2, 868	11.643	**<0.001**	0.026
Learning about species, environment, and science	2, 868	25.876	**<0.001**	0.056
**Skills (*n* = 859)**				
Gain in skills of data collection	2, 856	9.295	**<0.001**	0.021
Gain in skills of data analysis, etc.	2, 856	15.396	**<0.001**	0.035

(*p-values in bold are significant

**small effect: partial η^2^ ≥ 0.01, medium effect: partial η^2^ ≥ 0.06, large effect: η^2^ ≥ 0.14).

In contrast, the analysis of the respective coordinator data did not reveal any significant relationships between the kind or amount of information provided to the participants and participants’ perceived gains in knowledge or skills.

#### b. Training provided to and received by participants

**Fig 8:**
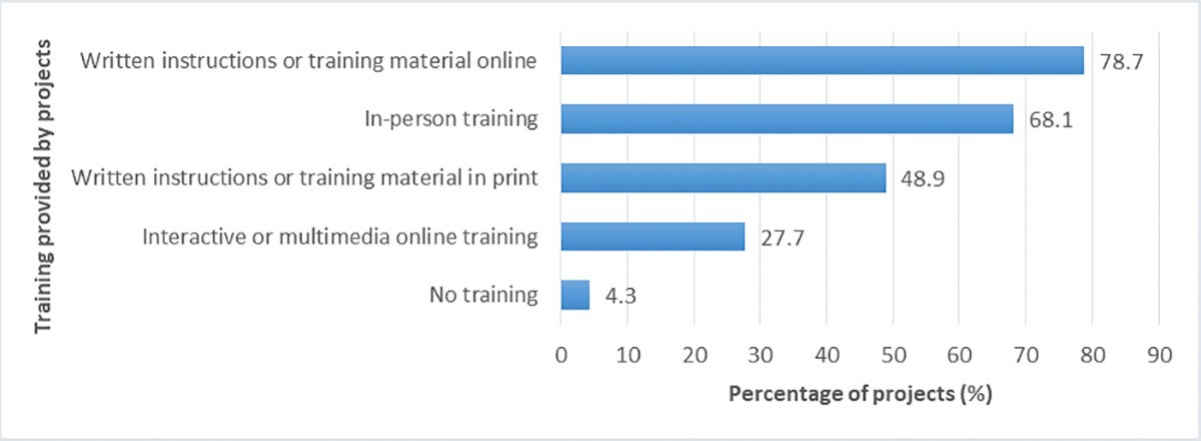
Training provided by the projects for their participants.

In the coordinator survey, the project staff was asked about the type of training they provided for their participants (multiple-choice question). The type of training most often provided was written instructions or training material online (79% of projects), followed by in-person training such as workshops and seminars (68%) (Fig 8).

**Fig 9:**
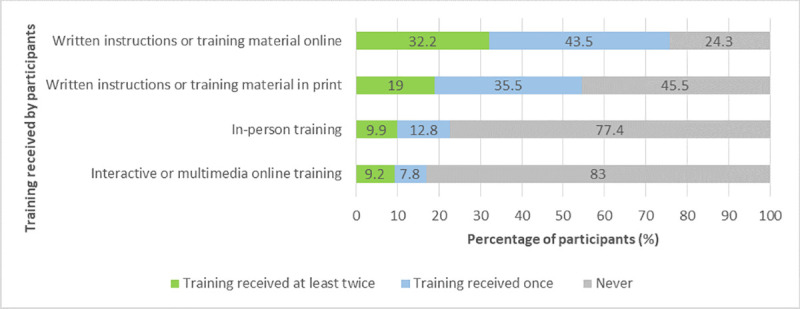
Training received by project participants.

Likewise, in the participant survey, participants were asked about the kind of training they received, and whether they received this training only once or at least twice. The training most often received by participants was written instructions or training material either online (76%) or in print (55%), followed by in-person training (23%) and interactive or multimedia online training (video, quiz, etc.) (17%) (Fig 9).

The analysis of the participant data indicated that participants’ perceived gains in knowledge and skills were significantly related to the amount of in-person or multimedia training they received; effect sizes were small (Table 6). Participants who received more in-person or multimedia training reported higher gains in knowledge and skills. No significant relationships were found for the amount of written instructions or training material that participants received.

**Table 6:** Results of the one-way independent ANOVA test for the amount of in-person training received by the project participants.

Scale	*df* (Degrees of freedom)	*F*	*p* (Significance)*	Partial η^2^ (Effect size)**
**Knowledge (n = 871)**				
Change in awareness of species	2, 868	7.452	**0.001**	0.017
Change in understanding of biodiversity	2, 868	11.518	**<0.001**	0.026
Learning about species, environment, and science	2, 868	15.134	**<0.001**	0.034
**Skills (n = 859)**				
Gain in skills of data collection	2, 856	10.353	**<0.001**	0.024
Gain in skills of data analysis, etc.	2, 856	20.977	**<0.001**	0.047

(*p-values in bold are significant

**small effect: partial η2 ≥ 0.01, medium effect: partial η2 ≥ 0.06, large effect: η2 ≥ 0.14).

The analysis of the coordinator data concerning training opportunities revealed significant results only for data collection skills: participants of projects that offered some kind of training reported higher data collection skills than participants of projects that did not offer any kind of training; effect sizes were small.

#### c. Opportunities for social interaction among participants

**Fig 10:**
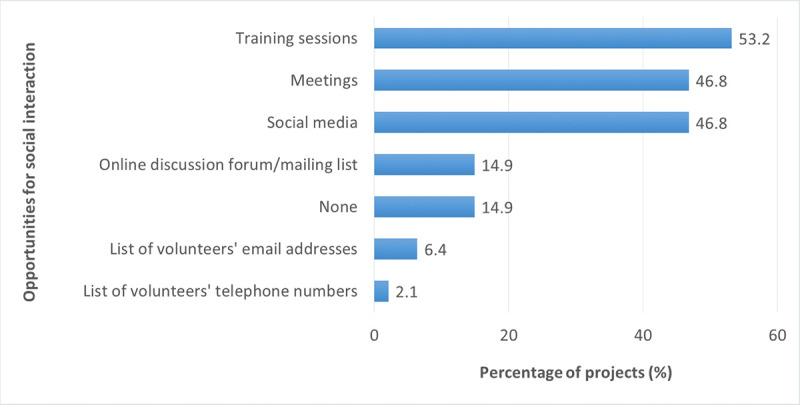
Opportunities for social interaction among participants as offered by the projects (n = 48).

In the coordinator questionnaire, respondents provided information on opportunities for social interaction that were available to participants (multiple-choice question). Most projects stated that training sessions offered opportunities for participants to interact (53% of projects); this was followed by social media (47%) and meetings (47%) (Fig 10).

Respondents to the participant questionnaire stated that they mostly used the following resources when contacting other volunteers (multiple answers possible): list of volunteers’ email addresses (15%), followed by meetings (14%), online discussion forums or mailing lists (11%), social media (10%), training sessions (9%), and a list of volunteers’ telephone numbers (6%). However, the majority of respondents (62%) had not been in contact with other participants yet, 24% had been in contact less than once a month, and 14% had been in contact at least once a month.

We also asked project participants whether they worked together with others when collecting data for the project. The majority of respondents (60%) worked alone, only 30% sometimes worked with others, and 10% always collected data with others. The respondents who worked together with others most often did so with family members, followed by other members of the same BDCS project, friends or neighbors, and members of a community club.

Our analyses of the participant data showed that participants’ amount of social interaction with other volunteers was significantly related to participants’ perceived gains in knowledge (with small effect sizes) and skills (with small to medium effect sizes) (Table 7): Participants who interacted more with other project participants reported higher gains in knowledge and skills. Furthermore, participants who worked with others when collecting data for the project reported higher gains in skills; effect sizes were small.

**Table 7:** Results of the one-way independent ANOVA test for whether participants had been in contact with other participants or not.

Scale	*df* (Degrees of freedom)	*F*	*p* (Significance)*	Partial η^2^ (Effect size)**
**Knowledge (n = 871)**				
Change in awareness of species	1, 869	14.803	**<0.001**	0.017
Change in understanding of biodiversity	1, 869	21.67	**<0.001**	0.024
Learning about species, environment, and science	1, 869	37.957	**<0.001**	0.042
**Skills (n = 859)**				
Gain in skills of data collection	1, 857	24.137	**<0.001**	0.027
Gain in skills of data analysis, etc.	1, 857	65.077	**<0.001**	0.071

(*p-values in bold are significant

**small effect: partial η2 ≥ 0.01, medium effect: partial η2 ≥ 0.06, large effect: η2 ≥ 0.14).

By contrast, our analyses of the coordinator data did not reveal any significant relationships between participant outcomes and opportunities for social interaction provided by the project.

#### d. Contact between project participants and project staff

**Fig 11:**
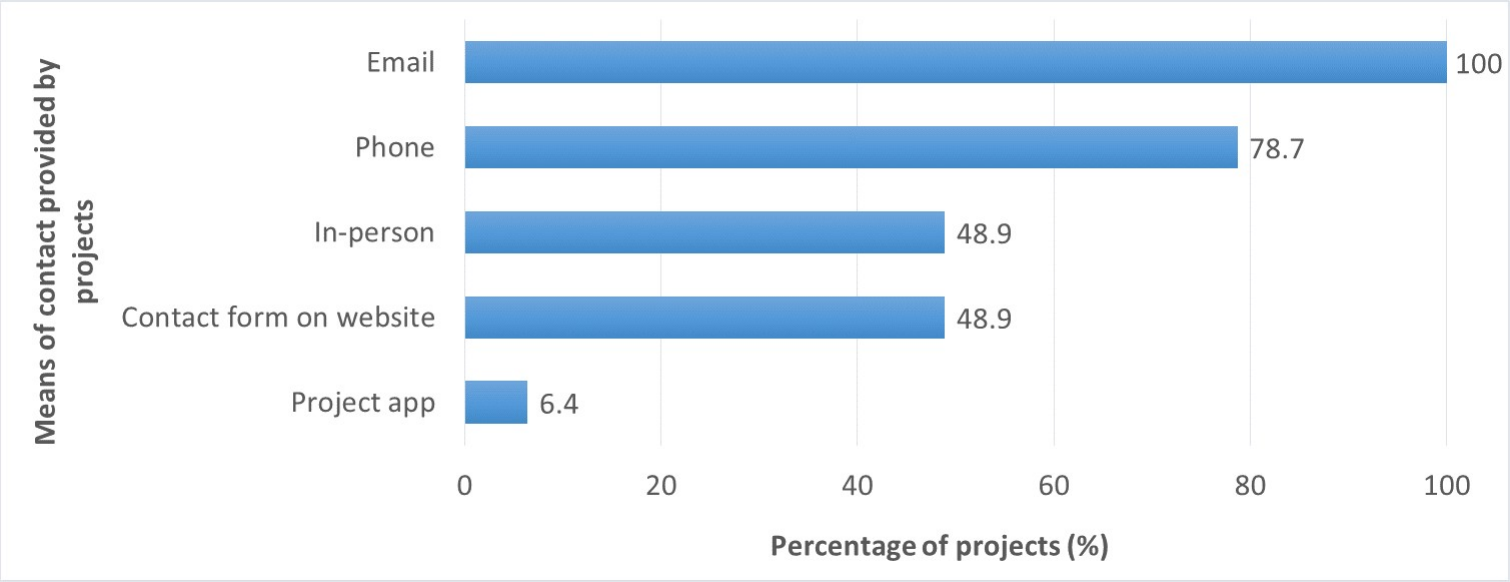
Means of contact with project staff provided by the projects.

In the coordinator questionnaire, respondents were asked to describe how project participants could contact project staff when they had questions or problems (multiple-choice question). All projects stated that their staff could be contacted via email; phone contact was offered by 79% of projects (Fig 11). When asked whether participants had the opportunity to meet the project scientists personally, 77% answered positively.

Project participants were asked how often they communicated with project staff through phone, email, online forum, and so forth. Nearly 30% of respondents stated that they never communicated with project staff. The majority of respondents communicated with staff less than once a month (61%); the remaining 10% communicated with staff once a month or more often. When asked how often they met the project scientists in person, 71% responded that they never met the scientists in person, 27% met the scientists less than once a month, and 2% met them at least once a month.

Analyzing the participants’ perspective, we found that the participants’ reported amount of contact with project staff was significantly related to perceived gains in knowledge (small effect sizes) and skills (small to medium effect sizes) (Table 8). The same applied to participants’ reported amount of contact with the project scientists in particular. Participants who had more contact with project staff and scientists perceived higher gains in knowledge and skills.

**Table 8:** Results of the one-way independent ANOVA test for the amount of contact between project participants and project staff.

Scale	*df* (Degrees of freedom)	*F*	*p* (Significance)*	Partial η^2^ (Effect size)**
**Knowledge (n = 840)**				
Change in awareness of species	4, 835	3.371	**0.01**	0.016
Change in understanding of biodiversity	4, 835	1.351	0.249	0.006
Learning about species, environment, and science	4, 835	4.207	**0.002**	0.020
**Skills (n = 828)**				
Gain in skills of data collection	4, 823	7.248	**<0.001**	0.034
Gain in skills of data analysis, etc.	4, 823	12.027	**<0.001**	0.055

(*p-values in bold are significant

**small effect: partial η2 ≥ 0.01, medium effect: partial η2 ≥ 0.06, large effect: η2 ≥ 0.14).

Analyzing the coordinators’ perspective, means of contact with project staff as provided by the project were not found to be connected to participant outcomes. Similarly, whether or not the projects provided their participants with the opportunity to meet the project scientists was not significantly associated with participant outcomes.

#### e. Feedback and recognition provided to and received by participants

We asked project coordinators whether they provided their participants with individual feedback on their performance of project tasks (e.g., on whether they identified a species correctly). The majority of projects sometimes provided individual feedback (55%), 13% regularly provided such feedback, and 11% of projects stated that participants received feedback every time they submitted data. 21% of projects did not provide participants with individual feedback.

**Fig 12:**
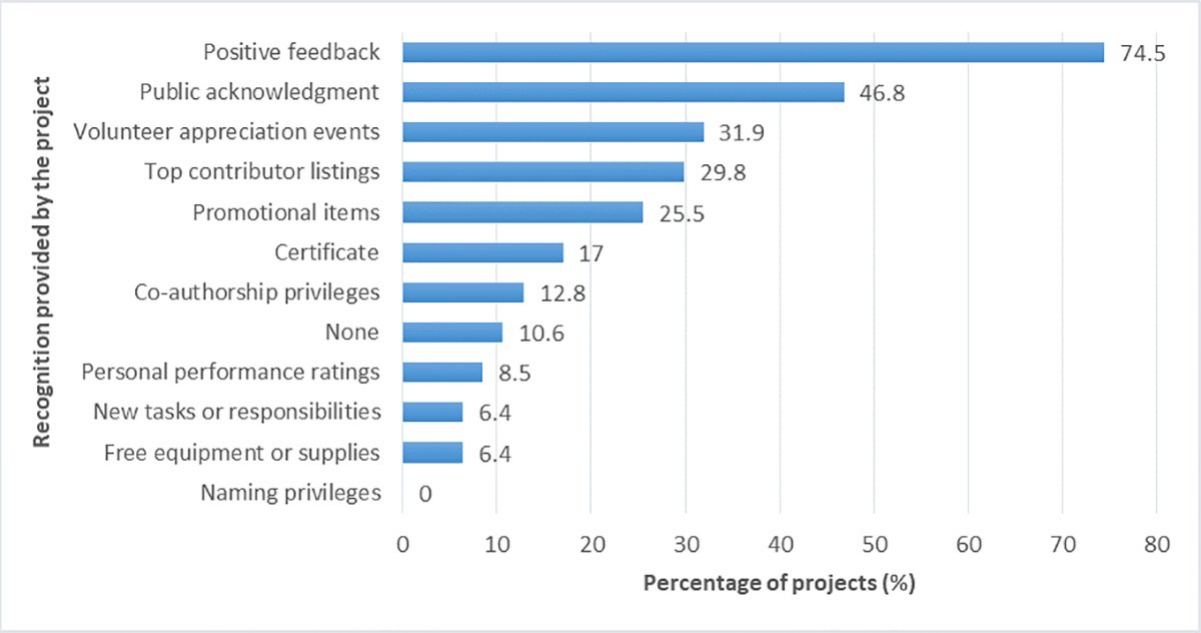
Forms of recognition or rewards provided by the projects.

When asked what kind of recognition or rewards the projects provided to volunteers for participating in the project (multiple-choice question), the answer most often chosen was positive feedback (74% of projects), followed by public acknowledgment (47%) and volunteer appreciation events (32%) (Fig 12).

Likewise, we asked participants about the individual feedback they received from the project regarding their performance of project tasks. Most participants reported that they had not received any feedback (46%). Fewer respondents received feedback sometimes (25%), regularly (14%), or every time they submitted data (15%).

Concerning the kind of recognition or rewards that participants received for participating in the project, most participants reported that they received either positive feedback as a form of recognition (46%) or no recognition or rewards at all (40%). Other forms of recognition were scarcely obtained: top contributor listings (8%), public acknowledgment (6%), certificates (5%), promotional items (5%), free equipment or supplies (5%), volunteer appreciation events (4%), new tasks or responsibilities (3%), personal performance ratings (2%), and naming and co-authorship privileges (1%).

Our analyses of the participant survey data revealed that the frequency of individual feedback that participants received was significantly related to participants’ perceived gains in knowledge (small effect sizes) and skills (small to medium effect sizes): participants who received feedback more frequently reported higher gains in knowledge and skills (Table 9).

**Table 9:** Results of the one-way independent ANOVA test for the frequency of individual feedback received by project participants.

Scale	*df* (Degrees of freedom)	*F*	*p* (Significance)*	Partial η^2^ (Effect size)**
**Knowledge (n = 871)**				
Change in awareness of species	3, 867	5.461	**0.001**	0.019
Change in understanding of biodiversity	3, 867	7.028	**<0.001**	0.024
Learning about species, environment, and science	3, 867	14.657	**<0.001**	0.048
**Skills (n = 859)**				
Gain in skills of data collection	3, 855	14.272	**<0.001**	0.048
Gain in skills of data analysis, etc.	3, 855	17.166	**<0.001**	0.057

(*p-values in bold are significant

**small effect: partial η2 ≥ 0.01, medium effect: partial η2 ≥ 0.06, large effect: η2 ≥ 0.14).

Recognition or rewards received by participants were also connected to participant outcomes: participants who received some kind of recognition reported significantly higher gains in knowledge and skills than participants who received no recognition at all (small effect sizes). Concerning specific forms of recognition, only positive feedback had significant effects: Participants who received positive feedback as a form of recognition reported significantly higher gains in knowledge and skills (with small effect sizes) than participants who did not receive this kind of recognition. Other kinds of recognition were not significantly related to participant outcomes.

Regarding data provided in the coordinator survey, we did not find clear relationships. The amount of individual feedback provided by the projects as well as the forms of recognition or rewards did not seem to be significantly associated with participant outcomes.

## Discussion

In our exploratory study, we investigated the extent to which participants’ gains in knowledge and skills were connected to the following project characteristics: information provided to participants, training provided to participants, opportunities for social interaction among participants, contact between project participants and project staff, and feedback and recognition provided to participants. We examined the perspectives of both project coordinators and project participants, that is, we looked at, for example, feedback provided by the project (as reported by project coordinators) and feedback received by the participants (as reported by project participants). While analyses of the participant survey data revealed connections between all of the above project characteristics and participants’ gains in knowledge and skills, analyses of the coordinator survey data yielded hardly any statistically significant results.

### Project characteristics from the participants’ perspective

Participants’ perceived gains in biodiversity-related knowledge and skills were significantly related to the project characteristics that were reported in the participant survey.

#### Information received by participants

We found that project participants who received more information on the project’s goals, the scientific background and results, the threats to the species the project focused on, and the opportunities for engaging in conservation activities outside the project reported higher gains in knowledge and skills. These results are supported by previous research on learning in CS projects. Haywood [52] found that CS project participants need to "understand the big picture" in which their data collection takes place. By comparing their own findings with the overall project results, participants get the opportunity to form links between local, regional, and possibly even global environmental issues [40]. Understanding this broader context contributes to science learning [84]. The need for CS projects to provide information that explicitly explains the project’s scientific background and processes is supported by Brossard et al. [85]. They found that participants’ content knowledge increased during project participation, but participants’ understanding of the scientific process did not. They reasoned that simply providing participants with reading material might not be sufficient. Instead, both Brossard et al. [85] and Pandya and Dibner [44] emphasized that learning about science would be increased if participants received explicit information not only about the content, but also about the research process in which they are involved. Wyler and Haklay [86] specifically recommended "full transparency of the research objectives, research protocol and analysis techniques" (p. 177). Overall transparency concerning all aspects of the project, including intended and achieved project outcomes, would clearly benefit participants’ gains in environmental and scientific knowledge and skills.

#### Training received by participants

In our study, project participants who took part in interactive or multimedia online training or in-person training reported higher gains in knowledge and skills. By contrast, written instructions or training material in both printed and online versions did not seem to have any connection with knowledge or skills. In a similar way, Garcia-Soto et al. [87] found that providing participants with photographic guides and text descriptions of species was not sufficient to ensure that participants correctly identified seagrass species, especially in the case of species that participants had not encountered before. Frequent misidentification of seagrasses was the result. Face-to-face training in the field turned out to be necessary for participants to gain the appropriate skills. Peltola and Arpin [68] also found that face-to-face training sessions outdoors had an important impact on participants’ learning. Furthermore, in-person training workshops led to participants’ increased environmental knowledge [70, 71], science knowledge [70], and scientific skills [70, 88]. Our findings are also supported by van der Wal et al. [72], who demonstrated that an interactive online training program increased participants’ species identification skills. Especially beginners appreciated the tool that helped them to acquire these skills that they perceived as difficult. We thus conclude that interactive online or in-person training is most effective. Jordan et al. [34], however, reported that a training workshop that consisted of providing content information and practicing species identification increased participants’ content knowledge, but not their understanding of the nature of science. The authors concluded that the training did not offer enough time and opportunities for practice and reflection. Participants need to reflect on their role in the scientific process, in order to achieve science learning outcomes [85, 89].

Thus, interactive training, be it in-person or online, needs to build in such opportunities for practice and reflection. One way of doing this might be by not only offering separate training sessions, but by actually integrating training directly into the project activities, as recommended by Edelson et al. [55]. This could be done, for example, by giving participants educational tasks as part of the data collection protocol [55]. In addition, Pandya and Dibner [44] suggest learning support in the form of, for example, mentoring of new participants by more advanced participants. This could serve as a kind of ongoing on-the-job training, enabling participants to reflect on and apply the skills and knowledge they have gained. In all this it is important to bear in mind that training of any kind needs to be adapted for the target audience and its needs. Kountoupes and Oberhauser [56], for example, reported that training activities were successfully modified in order to make them more suitable for younger participants by including more hands-on activities and "practicing rather than talking". We conclude that interactive forms of online or in-person training, if well integrated into the project and tailored to the participants’ needs, can effectively support participants in their learning.

#### Opportunities for social interaction among participants

We found that participants who interacted more with other project participants reported higher gains in knowledge and, especially, in skills. In addition, participants who collected data together with other people (most often, family members) reported higher gains in skills than participants who worked on their own. Our findings are supported by Peltola and Arpin [68], who found that social interaction in a group led to collective learning, which in turn resulted in increased knowledge and skills on the part of the project participants (also see Lave and Wenger [90]). More specifically, Deguines et al. [15] observed that the level of social interaction among project participants on the project’s website (e.g., through commenting on each other’s photographs) was positively related to participants’ gains in species identification skills. Similarly, a real-time message or chat function on the project website was found by Tinati et al. [59] to facilitate participants sharing knowledge and information and learning from each other.

The respondents of our participant survey who reported collecting data together with others most often did so with family members. Engaging families in CS projects could hold a potential for increased participation and learning. Kountoupes and Oberhauser [56] argued that since there is a general lack of informal environmental and science education programs for adults, involving their children in educational activities could be a motivator for parents to participate in CS projects. In the study by Evans et al. [62], project participants mentioned that they took part in the project because they wanted their children to learn about the environment. Through this shared or social learning, both parents and children could benefit by gaining knowledge and skills.

However, our findings indicate that social interaction in CS projects might not happen automatically. The majority of our participant survey respondents stated that they had not been in contact with any other participants; similarly, most stated that they collected data on their own. CS projects, therefore, need to be deliberately designed for social interaction, as emphasized by Davis et al. [61]. Such interaction needs to be actively encouraged, for example, by promoting data collection in pairs or teams, by having participants verify each other’s classifications [44], or by offering regular activities that specifically address families. Another potential form of social interaction was described by Davis et al. [61]: social data sharing events as part of an environmental health-related CS project. During these events, project participants who lived close to each other compared data that they had collected and discussed potential reasons for differences in their data. BDCS projects are often based on individuals collecting data. By encouraging social interaction among project participants, these projects can be designed in a way that makes their participants feel part of a larger endeavor [44] and that encourages learning within a community (see Wenger [91]).

#### Contact between project staff and participants

We found that project participants who had more contact with project staff and scientists reported higher gains in knowledge and, particularly, in skills. Evans et al. [62] even described interactions between research staff and project participants to be one of the two most important factors influencing learning. Participants in the study by Evans et al. [62] commented that meeting the project scientists face-to-face had the biggest influence on their knowledge and skills. Participants learned, for example, from observing the scientists, and also from discussions with the scientists. Participants who had the chance to directly interact with researchers felt empowered and appreciated as partners in the research process [62]. Koss and Kingsley [63] and Toomey and Domroese [65] also argued that direct interaction between scientists and participants influenced learning outcomes. Peltola and Arpin [68] mentioned long-term relationships between project participants and instructors as having contributed to participants’ learning.

Past research supports the results of our study that suggest that increased contact between project participants and project staff is connected to participants’ learning. However, in our study, the majority of participants reported that they had had little contact to project staff and had not met the project scientists yet. This indicates that there is a need for more intentional interaction. Edelson et al. [55] specifically recommended interaction between "science experts" and project participants as an important strategy for improving participants’ scientific skills and understanding of scientific concepts. Such interaction can take place, for instance, through ongoing participant training events [52] or visits to areas where participants regularly monitor species [62]. Davis et al. [61] suggest deliberately designing CS projects for dialogue and interaction, for example, through in-person training events that can also serve as participant recruitment events, weekly conference calls with project staff and key volunteers, annual professional development events with project staff and key volunteers, and "regular opportunities for open, friendly engagement between project staff, […] and participants at community trainings, data sharing events, and informal ’open house’ events throughout each year" (p. 20). Several authors have emphasized the importance of intentional project design for achieving participant outcomes (e.g., [36, 44, 74, 89]. By deliberately designing BDCS projects to include interaction among project participants, staff, and scientists, these projects could potentially achieve higher learning outcomes for the participants.

#### Feedback and recognition received by participants

In our study, project participants who frequently received individual feedback from the project regarding their performance of project tasks reported higher gains in knowledge and, especially, in skills. Similarly, participants who received positive feedback as a form of recognition or reward for participating in the project reported higher gains in knowledge and skills. Previous research on BDCS projects confirms these findings. BDCS projects, such as the ones surveyed in our study, often involve participants in collecting specimens (or taking photographs thereof), identifying the specimens, and submitting them (or the photographs thereof) to the project. Druschke and Seltzer [36] described that, in their CS project, "participants did not get feedback about the species of bees they actually collected or whether they collected any bees at all" (p. 182). The authors saw this as a missed opportunity to educate their participants about pollinators, and they advised future projects to report back to the participants as quickly as possible about the collected or identified species. As a result, participants would feel that their contribution is valued and that they are part of the overall research process [36]. Van der Wal et al. [72] demonstrated that immediate automated feedback upon data submission allowed project participants, especially beginners, to quickly gain or expand their species identification skills. This kind of feedback even motivated the participants to further improve their identification skills. The authors argued that, because recording species is often a rather solitary activity, which was confirmed by the 60% of our survey respondents who collected data on their own, individual feedback to participants is particularly valuable for participants’ learning. Pandya and Dibner [44] agree that immediate feedback to project participants regarding the accuracy of submitted data can enhance participants’ content knowledge and scientific skills. Peltola and Arpin [68] confirm that giving regular positive feedback to project participants can help the participants to become more confident in their abilities and can thereby contribute to their learning (also see Hattie and Timperley [92]).

We conclude that participants would benefit from, on the one hand, regular and rapid individual feedback on the accuracy of the data they have collected and submitted, and on the other hand, from recognition of their participation in the project through positive feedback. Edelson et al. [55] recommend giving participants positive feedback by publicly acknowledging their contribution to the project. In addition, Pandya and Dibner [44] suggest that general feedback could include information on how the collected data were used by scientists in the past, and on how data have been and can be used to support decision making and inform policy in the future. By providing appropriate feedback to participants, BDCS projects can assist their participants in gaining knowledge and skills.

### Project characteristics from the project coordinators’ perspective

In contrast to the results discussed above, gains in knowledge and skills perceived by the participants were scarcely connected to the project characteristics reported in the coordinator survey. No statistically significant connections were found for information provided by the project, opportunities for social interaction among participants offered by the project, opportunities for contact with project staff and scientists offered by the project, or feedback and recognition provided by the project. Only in the case of training provided to the participants did we find that participants of projects that offered some kind of training reported higher data collection skills than participants of projects that did not offer any training. There seems to be a general discrepancy between participant and coordinator perspectives.

There are several possible reasons for this discrepancy between the results obtained from analyzing participant data and coordinator data. First, it is possible and even likely that certain project characteristics are perceived differently by project coordinators and participants. For example, in the case of information: projects might provide certain information, but participants might not receive it. If information is provided on the project website, only those participants who actively visit the website and navigate to those pages that contain the relevant information will perceive that they received the respective information. Information provided in the form of emails or postal mail might have better chances of being received by participants, but they still have to open the (e)mail, scroll through it, and read the information. Thus, some effort on the part of the participant is required in order to receive the information offered by the project. Participants who do not make that effort might simply not have noticed that the information was provided. The same might apply to opportunities for social interaction among participants, contact between project staff and participants, and training opportunities. These opportunities might have been provided by the project, but that did not automatically mean that participants made use of them. Only participants who made the effort and chose to use the opportunities offered by the project will have stated in the participant questionnaire that they received certain training or were in contact with project staff or scientists. More generally speaking, project participants might engage in their project and use certain project features in ways that were not necessarily intended by the project. Or as Edwards et al. [43] put it, "people learn different things in different ways within the same project. In other words, how learning is designed into citizen science projects does not guarantee that volunteers will learn what is intended or in the ways planned" (p. 388). This leads to potential methodological conclusions for our current and also for potential future research: in the case of certain project characteristics, the inherent difference between features provided by the project and features received and used by the participants might make it impossible to infer one from the other. It could therefore be assumed that certain project characteristics as described by the project coordinators cannot be directly connected to potential participant outcomes. In such cases, statistically significant relationships can only be found when project characteristics that are actually received by the participants (and this is indicated in their perception) are investigated.

Another reason for the observed discrepancy between project characteristics as reported by project coordinators and project participants might be the flow of information. Projects might offer a variety of training opportunities or means of getting in direct contact with other participants or with project staff and scientists, but participants might simply not know about these opportunities and, thus, might not make use of them. Again, this could be due to participants not having made the effort to read the respective information given by the project, as described above. But it could also be due to the way in which information is provided to the participants and the question of whether this way of providing information is suitable for the audience. Depending on whom the project wants to reach, the means of communication might have to be adapted to the audience. From the coordinator survey we know that information was provided by projects primarily on the project website and through emails. These forms of communication might be easiest to manage, but other forms might be more effective in reaching the target audience. A postcard in the postal mail could be more suitable for reaching older participants, while a message through a smartphone app might be more effective in addressing younger participants. In addition, Santori et al. [41] suggest creating more direct links between the different means of communication. By linking, for example, the project website to social media and scientific publications, projects might be more successful in reaching their participants. In particular, reaching younger audiences should be high on the list of projects’ priorities. As the demographic profile of our participant survey indicates, the majority of participants in BDCS projects is 60 years and older. In the context of the decline in species experts [8], recruiting youths and young adults into the projects is an urgent task. Suitable modes of communication can play a crucial role.

Furthermore, certain project characteristics described by the project coordinators might apply to only a small number of participants. In-person training, for instance, which we found to be connected to participants’ learning, might only be offered in certain regions or for a small number of participants, and might therefore not be accessible or attractive to the majority of participants. Another example is the opportunity for project participants to meet the project scientists personally. Evans et al. [62] described this as being a key factor that influences participants’ learning. The majority of projects in our study stated that they offered this opportunity to their participants. However, coordinators’ comments revealed that these opportunities were often limited to an "annual conference" or "annual meeting", "twice yearly", "a species monitoring day once every two years", "when the results are released", or "can be arranged if really necessary". This might explain why 71% of the participant survey respondents stated that they had never met the project scientists in person, and only 2% had met them at least once a month. This means that there could be a great potential for increasing participants’ learning by providing more opportunities for personal interaction between project scientists and project participants.

Having said this, for many projects, increased interaction between professional scientists and volunteers might be difficult to achieve due to a lack of time and money. One project coordinator commented: "Our scientists are time-poor and are only invited to corporate learning days or large events where high level government personnel will be attending." Ways of participant-scientist interaction have to be found that are beneficial not only for the participants, but also for the scientists. This might be achieved, for example, by asking participants for feedback on the project, its procedures, and its results. Haywood [52] suggested that asking for feedback should go beyond a simple "what can we do to improve your experience?" question. Instead, participants could be involved in refining data collection processes and participant training by answering more specific questions such as "what can we do to improve the science we are developing, or the methods by which we collect information?" (p. 258). By asking for participants’ feedback, CS projects could also increase their participants’ engagement in the project [36, 37, 52]. Additionally, in-person contact with the project scientists could be complemented by electronic forms of communication. Wyler and Haklay [86] suggest, for example, blogs by the project scientists, electronic chats, and face-to-face video discussions allowing participants to ask questions and comment on the project. Recognizing the importance of contact between project staff and project participants, Wyler and Haklay [86] recommend employing, for instance, a community manager, who could promote and organize such scientist-participant interactions. They propose including such staff positions in the application for project funding. As a result, it might be possible to foster interactions among scientists and participants that are beneficial for both parties.

### Limitations and future research

Our study was conducted to explore the connections between BDCS project characteristics and participants’ perceived gains in knowledge and skill. The large scale of our study, which comprised 48 BDCS projects and 1,067 project participants, allowed us to gain insights into a broad range of projects and to draw valuable conclusions. Yet, this study has limitations, which future research could overcome.

First, as discussed by Peter et al. [17], project participants’ gains in knowledge and skills refer to gains as perceived and reported by the participant survey respondents themselves. Future research would benefit from study designs and methods that measure and assess actual gains in a more objective way. If possible, such assessments should be performed both before and after the participants take part in the project. In this way, it would be possible to compare pre-, post- and follow-up responses. So far, these kinds of studies have rarely been conducted due to the difficulty of implementing them in CS projects, which rely on participants taking part in the project voluntarily [45]. Embedded assessment as suggested by, for example, Becker-Klein et al. [93] could be an alternative.

In a similar way, our study was based on project characteristics as reported by the project coordinators and project participants. In addition to reported information, it might be beneficial to objectively measure project characteristics where possible. This might be feasible for project characteristics that are easy to measure and record, such as the amount of social interaction among participants on an online platform, the level of online interactive training that participants take part in, or the kind of automated online feedback that participants receive (see, e.g., van der Wal et al. [72], Aristeidou et al. [94, 95]). However, a direct investigation of project characteristics and their connection to participants’ learning might be limited to the aspects of a project that take part online. Mixed-methods approaches might be able to examine online and offline aspects of CS projects and capture project characteristics from different perspectives.

Another potential limitation of our study could be the different size of the CS projects in our study and, hence, the number of project participants responding to the participant survey. Large projects with a high number of survey participants might have had a disproportionately large influence on the results obtained from analyzing the data provided by the coordinators. Conversely, projects with few participant survey respondents might have had a disproportionately small influence on the analysis of the coordinator data. Study results might be improved by incorporating more projects into a comparative study. This would reduce the impact of individual projects on the analysis. In addition, case studies of single (preferably large) CS projects could focus on specific project characteristics in more depth. Ideally, such studies would include control groups for the project characteristics of interest. Such a study could, for example, compare gains in the data collection skills of participants who received either no training, printed species identification brochures only, an interactive online training course, or an in-person training session in the field. In-depth studies could also comprise qualitative methods such as interviews and focus groups to gain more comprehensive insights into the connections between project characteristics and participants’ learning outcomes.

The findings of our exploratory study can provide a basis for in-depth research on CS project characteristics and their connection to participants’ gains in knowledge and skills. Yet, future research should focus not only on the connection to knowledge and skills, but also on the connection to other participant outcomes such as gains in interest, motivation, and self-efficacy, and changes in behavior (see, e.g., Phillips et al. [30], Peter et al. [17]). Moreover, further research on project characteristics that were not addressed in our study would be valuable. Such characteristics could be, for example, the level of difficulty of project activities [35, 52], levels or layers of participation that are available to volunteers [18, 37, 52], the availability and accessibility of data to project participants [96, 97], or characteristics of a survey site in connection with participants’ attachment to place [40, 62].

## Conclusion

In our study, we explored characteristics of BDCS projects. While existing CS literature mainly focuses on the connection of project design to general project success or to overall learning outcomes, we specifically addressed the connection to participants’ biodiversity-related knowledge and skills. We conducted a comprehensive study across 48 BDCS projects in 10 countries. Our study included the perspective of both the project coordinators and the project participants. In this way, our study is the first to systematically investigate a number of specific project characteristics and their connection to participants’ gains in knowledge and skills.

The results of our research suggest that participants’ perceived gains in knowledge and skills were positively associated with the information they received from the project, the training they took part in, the amount of interaction they had with other project participants, the amount of contact they had with project staff and scientists, and the feedback and recognition they received from the project. This indicates that it is important to deliberately integrate these features into the design of CS projects if participants’ learning is among the project goals.

The importance of intentional project design for achieving participant outcomes has been emphasized in the past. With our present study, we provide insights into project characteristics that can impact participant outcomes. We hope that our findings will contribute to an improved design of BDCS projects. As a result, BDCS projects could be more effective in fostering participants’ gains in knowledge and skills. By improving participants’ knowledge as well as their awareness and understanding of biodiversity and by increasing participants’ skills in identifying biodiversity, BDCS projects can support the conservation of biological diversity on Earth.

## Supporting information

S1 File S1–S13 Tables. (PDF)
