# Supplementary material for: Citizen science project characteristics: Connection to participants’ gains in knowledge and skills
Source: PLoS One. 2021 Jul 15;16(7):e0253692. doi: 10.1371/journal.pone.0253692 (PMC8282002; doi:10.1371/journal.pone.0253692)
Supplement: S1 File — (PDF) [file pone.0253692.s001.pdf]

S1 File

SUPPORTING INFORMATION

Article

Citizen science project characteristics: Connection to participants' gains in knowledge and skills

Authors

Maria Peter, Tim Diekötter, Tim Höffler, Kerstin Kremer

A. Citizen science projects participating in the study

Table S1. Biodiversity citizen science projects included in our study (sorted alphabetically by country and project name).

Country	Project name	Coordinating organization
Australia	Batwatch/MEGA Murray-Darling Microbat Project	Natural Resources South Australia Murray-Darling Basin, and partners
Australia	Birdwatch/Community Bird Monitoring	Natural Resources South Australia Murray-Darling Basin
Australia	Canberra Nature Map	Private (syndicate of volunteers)
Australia	ClimateWatch	Earthwatch Institute Australia
Australia	DuneWatch	Griffith University Centre for Coastal Management
Australia	FrogWatch South Australia	FrogWatch South Australia/Green Adelaide
Australia	New South Wales Bird Atlas	New South Wales Bird Atlassers Inc.
Australia	Reef Life Survey	Reef Life Survey Foundation
Australia	Sea Slug Census	Southern Cross University, and local partners
Australia	Wild Pollinator Count	Wild Pollinator Count
Austria	Amphibien und Reptilien Österreichs unter Beobachtung	Naturhistorisches Museum Wien
Austria	Biodiversitätsmonitoring mit LandwirtInnen - Wir schauen auf unsere Wiesen und Almen	Österreichisches Kuratorium für Landtechnik und Landentwicklung (ÖKL)
Austria	Naturbeobachtung.at	Naturschutzbund Österreich
Austria	Pilzfinder.at	Österreichische Mykologische Gesellschaft
Austria	Schmetterlinge Österreichs	Blühendes Österreich - REWE International gemeinnützige Privatstiftung
Austria	Vielfalt bewegt! Alpenverein von Jung bis Alt	Österreichischer Alpenverein
Austria	Viel-Falter	Universität Innsbruck
Denmark	NaturTjek (Biodiversitet Nu)	Danmarks Naturfredningsforening
Finland	Butterfly Monitoring Scheme for Finland	Finnish Environment Institute

Country	Project name	Coordinating organization
Germany	ArtenFinder	Stiftung Natur und Umwelt Rheinland-Pfalz
Germany	Kerbtier.de - Käferfauna Deutschlands	Private (Christoph Benisch)
Germany	NABU|naturgucker	naturgucker.de gemeinnützige eG
Germany	Ornitho.de	Dachverband Deutscher Avifaunisten
Germany	StadtWildTiere Berlin	Leibniz-Institut für Zoo- und Wildtierforschung
Ireland	Countryside Bird Survey	BirdWatch Ireland
Ireland	Irish Garden Bird Survey	BirdWatch Ireland
Ireland	Irish Wetland Bird Survey	BirdWatch Ireland
Netherlands	Dutch Butterfly Monitoring Scheme - Meetnet vlinder	Vlinderstichting (Dutch Butterfly Conservation)
Sweden	Swedish Fauna Watch - Faunaväkteriet småkryp	Swedish Entomological Society, and partners
Switzerland	Atlas de la Flore Vaudoise	Cercle vaudois de botanique
Switzerland	Flora des Kantons Zürich	Zürcherische Botanische Gesellschaft
Switzerland	Ornitho.ch	Schweizerische Vogelwarte
Switzerland	StadtWildTiere Schweiz	Verein StadtNatur
UK	BeeWalk	Bumblebee Conservation Trust
UK	BirdTrack	British Trust for Ornithology
UK	Breeding Bird Survey	British Trust for Ornithology
UK	Bromley Swift Survey	Royal Society for the Protection of Birds Local Group Bromley
UK	Dragonfly Watch	British Dragonfly Society
UK	English Winter Bird Survey	British Trust for Ornithology
UK	Garden Bird Feeding Survey	British Trust for Ornithology
UK	Garden Bird Watch	British Trust for Ornithology
UK	Mammal Mapper - National Mammal Atlas Project	Mammal Society
UK	National Bat Monitoring Programme	Bat Conservation Trust
UK	Riverfly Recording Schemes	Buglife
UK	Rothamsted Insect Survey Light-trap Network	Rothamsted Research Centre
UK	UK Ladybird Survey	UK Centre for Ecology and Hydrology, and partners
UK	UK Wetland Bird Survey	British Trust for Ornithology, and partners
UK	Wider Countryside Butterfly Survey	Butterfly Conservation

B. Coordinator survey

Table S2. Coordinator survey. Questionnaire item for information provided to project participants.

**Do you provide your participants with information about the following?**								
	No	Yes, on the project website	Yes, through social media	Yes, mess-ages through the project app	Yes, through emails	Yes, through mailings (paper)	Yes, in training sessions	Other
Overall objectives / goals / intended outcomes of your project								
Scientific background and processes of your project								
Overall results / outcomes of your project								
Threats to the species that your project focuses on								
Opportunities for engaging in conservation activities outside your project								

Table S3. Coordinator survey. Questionnaire item for training provided to project participants.

**What type of training does your citizen science project provide for the participants?** Please choose all that apply.	
None	
Written instructions or training material online	
Written instructions or training material in print	
Interactive or multimedia online training (video, quiz, etc.)	
In-person training (workshop/seminar) - voluntary	
In-person training (workshop/seminar) - mandatory	
Other:	

Table S4. Coordinator survey. Questionnaire item for opportunities for social interaction among participants.

**What opportunities for social interaction with other volunteers are available to participants?** Please choose all that apply.	
None	
Online discussion forum or list serve / mailing list	
Social media (e.g., Facebook, Twitter, etc.)	
List of volunteers' email addresses	
List of volunteers' telephone numbers	
Meetings	
Training sessions	
Other:	

Table S5. Coordinator survey. Questionnaire items for contact between project participants and project staff and scientists.

**How can your participants contact project staff when they have questions or problems?** Please choose all that apply.	
Contact form on the project website	
Message through the project app	
Email	
Phone	
In person	
Other:	
**Do you provide your participants with the opportunity to meet the project scientists personally?** Please choose one of the following.	
No	
Yes (please specify):	

Table S6. Coordinator survey. Questionnaire items for feedback and recognition provided to participants.

**Do you provide your participants with individual feedback on their performance of project tasks (e.g., on whether they identified a species correctly)?**<br>Please choose one of the following.	
No, participants do not receive individual feedback	
Yes, sometimes	
Yes, regularly	
Yes, they receive feedback every time they submit data	
Other:	
**What kind of recognition or reward do volunteers receive for participating in the project?**<br>Please choose all that apply.	
None	
Positive feedback	
Free equipment or supplies	
Certificate	
Promotional items, e.g., stickers, pins, t-shirts	
Top contributor listings	
Personal performance ratings	
Public acknowledgment	
New tasks or responsibilities	
Naming privileges	
Co-authorship privileges	
Volunteer appreciation events	
Other:	

C. Participant survey

Table S7. Participant survey. Questionnaire items for various activities concerning information received, social interaction among participants, and contact with project staff and scientists.

**As part of your project, approximately how often do you do the following activities?**								
	Never	Only once so far	Once a year	A few times a year	About once or twice a month	About once a week	A few times a week	Every day
Read project newsletters or other information received from the project								
Communicate with other participants (phone, email, online forum, etc.)								
Communicate with project staff (phone, email, online forum, etc.)								
Meet the project scientist(s) in person								

Table S8. Participant survey. Questionnaire item for information received by project participants.

**Has your project provided you with information about the following?**			
	No	Yes, some information	Yes, comprehensive information
Overall objectives and goals of your project			
Scientific background and processes of the project			
Overall results of your project			
Threats to the species that the project focuses on			
Opportunities for engaging in conservation activities outside your project			

Table S9. Participant survey. Questionnaire item for training received by project participants.

**What kind of training did you receive in order to participate in your project?**			
	Did not receive this kind of training	Only once, when starting to participate	At least twice
Written instructions or training material in print			
Written instructions or training material online			
Interactive or multimedia online training (video, quiz, etc.)			
In-person training (workshop/seminar)			
Other training			

Table S10. Participant survey. Questionnaire items for opportunities for social interaction among participants.

**When you collect data for your project, do you work with others (in a pair or in a group)?** Please choose one of the following.	
No, never	
Yes, sometimes	
Yes, always	
**When you work with others, with whom do you collect data for your project?** Please choose all that apply.	
Family members	
Friends, acquaintances, or neighbors	
Community club	
Other members of the same citizen science project	
**What resources do you use when you want to contact other volunteers?** Please choose all that apply.	
List of volunteers' telephone numbers	
List of volunteers' email addresses	
Online discussion forum or list serve / mailing list	
Social media (Facebook, Twitter, etc.)	
Meetings	
Training sessions	
I have not contacted other volunteers yet	
Other:	

Table S11. Participant survey. Questionnaire items for feedback and recognition received by participants.

**Have you received any feedback on your performance of project tasks (e.g., on whether you identified a species correctly)?**<br>Please choose one of the following.	
No, I have not received any feedback yet	
Yes, sometimes	
Yes, regularly	
Yes, I receive feedback every time I submit data	
**Have you received any recognition or reward for participating in the project?**<br>Please choose all that apply.	
None	
Positive feedback	
Free equipment or supplies	
Certificate	
Promotional items, e.g., stickers, pins, t-shirts	
Top contributor listings	
Personal performance ratings	
Public acknowledgment	
New tasks or responsibilities	
Naming privileges	
Co-authorship privileges	
Volunteer appreciation events	
Other:	

Table S12. Participant survey. Questionnaire items for perceived changes in knowledge.

**Environmental and science knowledge**<br>Please indicate how much you agree or disagree with the following statements.					
"Participating in this project has made me more aware of …"					
	Strongly disagree	Disagree	Neutral	Agree	Strongly agree
…species' presence and behavior.					
…the principal threats to these species.					
…the diversity of species that exist in my region.					
Biological diversity (or biodiversity) is (among other things) the diversity of species that exist in an area. *"Participating in this project has increased my understanding of … "*					
	Strongly disagree	Disagree	Neutral	Agree	Strongly agree
…the term 'biological diversity' or 'biodiversity'.					
…the importance of biological diversity.					
…the threats to biological diversity.					
"As a result of participation in this project, I've learned a lot about …"					
	Strongly disagree	Disagree	Neutral	Agree	Strongly agree
…the species I have found or observed.					
…the environment and nature in general.					
…how science works.					

Table S13. Participant survey. Questionnaire items for perceived changes in skills (adapted from Phillips, T., Porticella, N., and Bonney, R., 2017. Skills for Science Inquiry Scale. Technical Brief Series. Cornell Laboratory of Ornithology, Ithaca).

**Skills and competences**<br>Please indicate how much you agree or disagree with the following statements.					
"Through participating in the project I have gained or improved the skills necessary to …"					
	Strongly disagree	Disagree	Neutral	Agree	Strongly agree
…observe/record species.					
…identify different species.					
…collect data in a standardized manner.					
…submit my observations to the project database.					
…use the project database to answer a question.					
…interpret the meaning of project data presented in maps, charts, graphs, etc.					
…conduct statistical analyses using project data.					
…design my own study related to project data.					
…communicate project findings to others.					
…train others to participate in the project.					
